# Supplementary material for: Parallelizing analog in-sensor visual processing with arrays of gate-tunable silicon photodetectors
Source: Nat Commun. 2025 May 21;16:4728. doi: 10.1038/s41467-025-60006-x (PMC12095749; doi:10.1038/s41467-025-60006-x)
Supplement: Supplementary file 1 — Supplementary Information [file 41467_2025_60006_MOESM1_ESM.pdf]

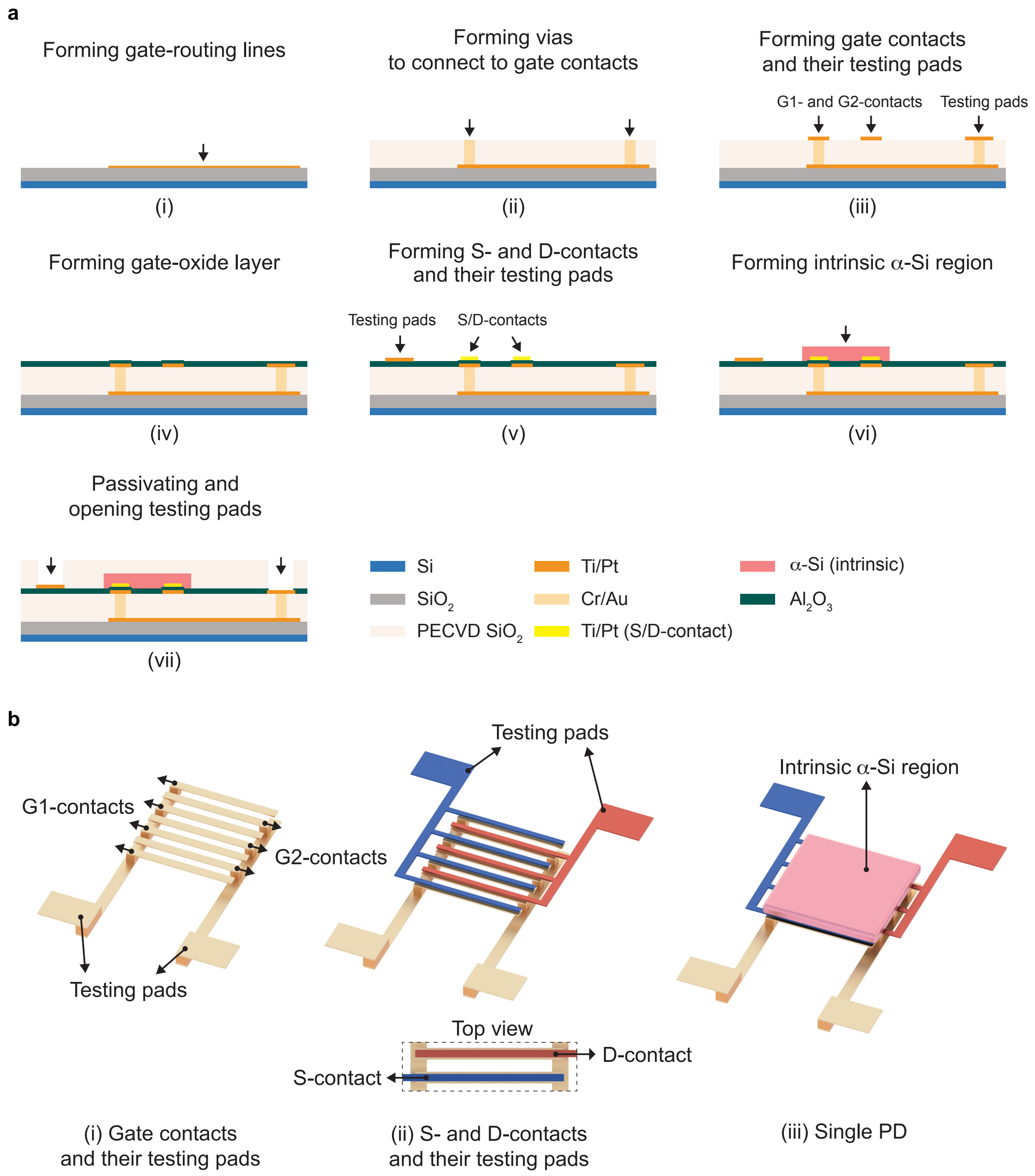

Supplementary Figure 1. Fabrication of individual PDs. a, Fabrication flow. **b**, 3D schematics.

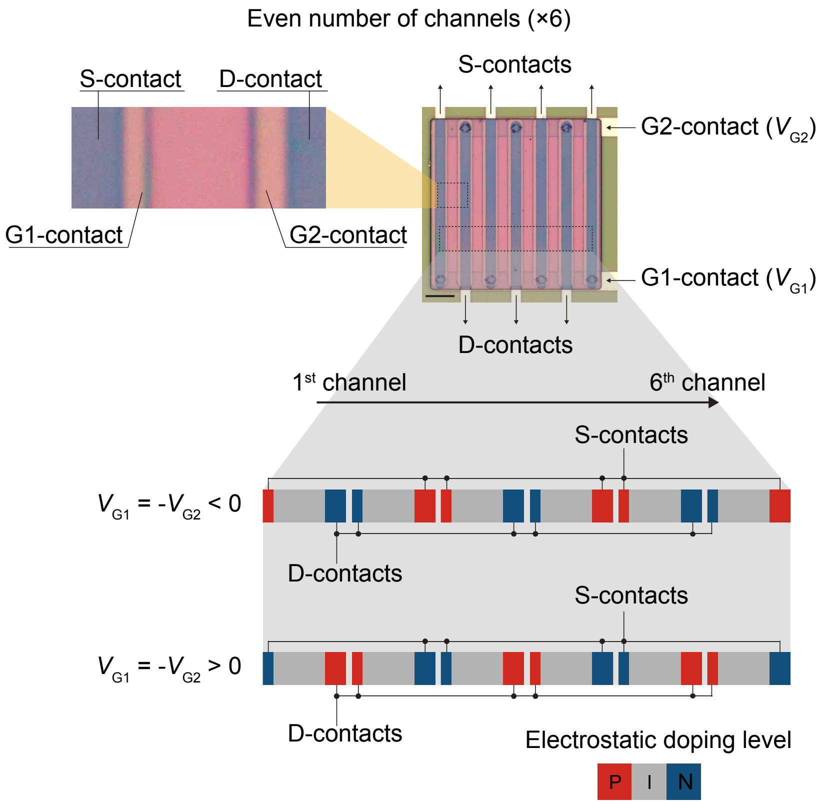

Supplementary Figure 2. PDs with an even number of channels have a less effect of alignment error on their photoresponse. The alignment error of S/D-contacts (e.g. the S-[D-] contact is accidentally formed closer [further] to the G1[G2]-contact of the 1st channel) brings asymmetry of the p- and n-doped areas in each channel. Such asymmetry in an even number of channels cancels each other when $V_{G1} = -V_{G2}$, leading to a symmetric $V_p - I_{ph}$ curve in Fig. 1**d**. Scale bar, 10 μm.

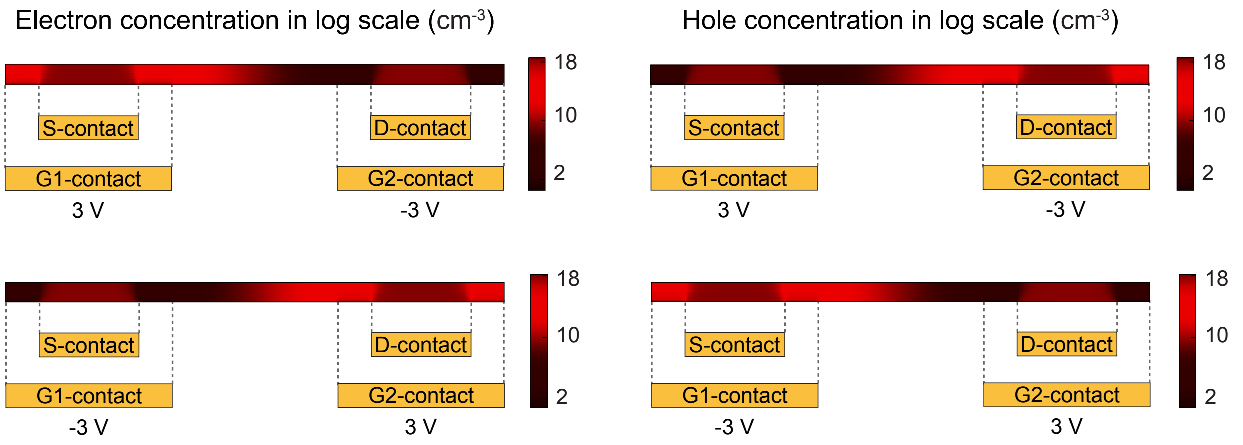

Supplementary Figure 3. COMSOL simulation of electron and hole concentration profiles (log scale) in short-circuited PDs.

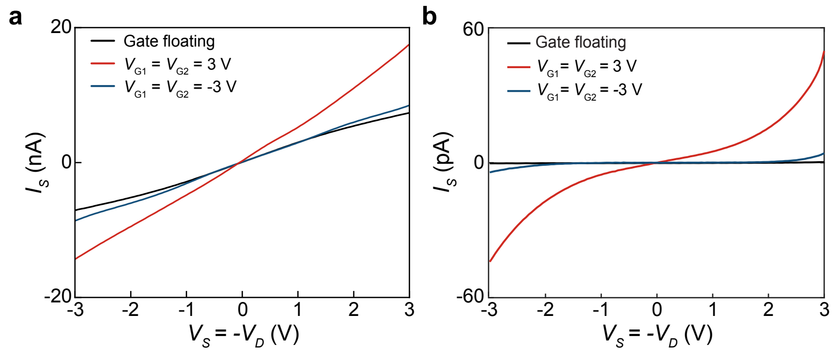

Supplementary Figure 4. Additional experiments on the optoelectronic characteristics of individual PDs. I_S - V_S curves of a representative PD when $V_{G1} = V_{G2}$ = -3 or 3 V and that when both G1- and G2-contacts are floated measured with P_{light} = 530 mW cm^{-2} at 550/15 nm (**a**) and in the dark (**b**).

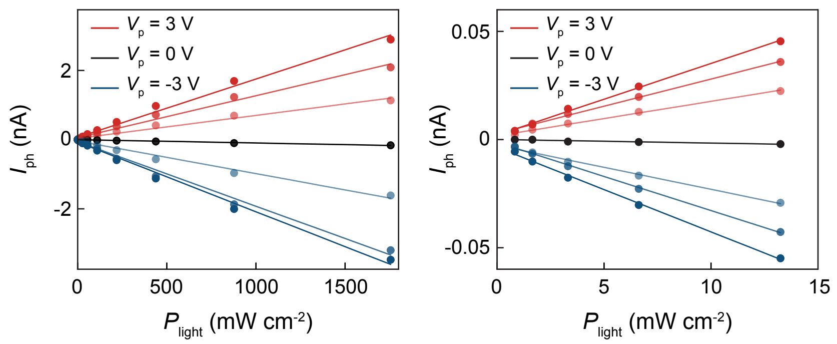

Supplementary Figure 5. Additional experiments on the linearity of individual PDs. I_{ph}-P_{light} curves and their zoom-in view obtained from another PD with V_p ranging from -3 to 3 V at a 1 V step, P_{light} = 0.83 - 1696 mW cm^{-2} at 550/15 nm, suggesting a *ca.*10^3 dynamic range with good linearity ($R^2 > 0.98$).

Note: This specific experiment is conducted differently from that in Fig. 1**e**. To quantify the dynamic range, weak P_{light} is provided by applying up to 512-time attenuation in the light path of the microscope via neural-density filters. To measure small-valued I_{ph} under weak P_{light}, the PD (wirebonded on the loading PCB) has its gate biases offered by the gating PCB, its D-contact biased at 0 V (via SMU), and its S-contact connected to the positive input of the TIA. The negative input of TIA is biased at 0V to amplify the short-circuited I_{ph} to V_{out} values (low-noise mode, gain = 2×10^9 V A^{-1}), which are then filtered by the noise eliminator and sampled by the digital oscilloscope at 10 kHz.

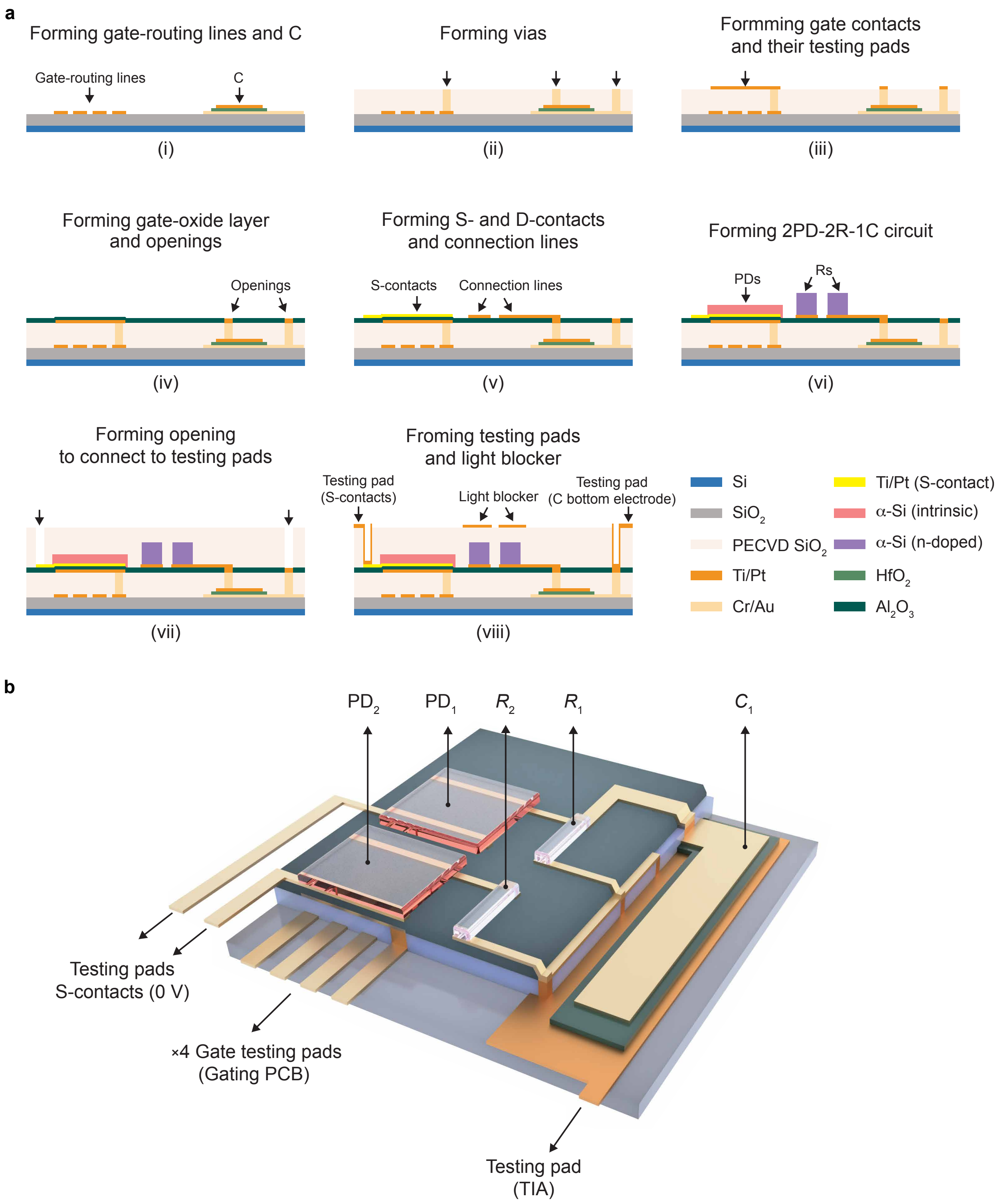

Supplementary Figure 6. Fabrication of single CUs. a, Fabrication flow. **b**, 3D schematics.

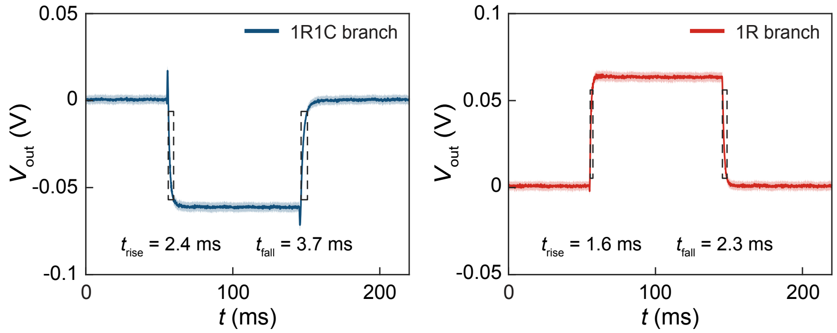

Supplementary Figure 7. V_{out}-traces from the 1R1C and the 1R branch of a single CU. The light spot (pulsing with ΔP_{light} = 530 mW cm^{-2} at 550/15 nm, same in Fig. 2**b**) is spatially confined to the PD in the select branch. Shaded areas represent ±1 S.D. from a total of 60 pulses.

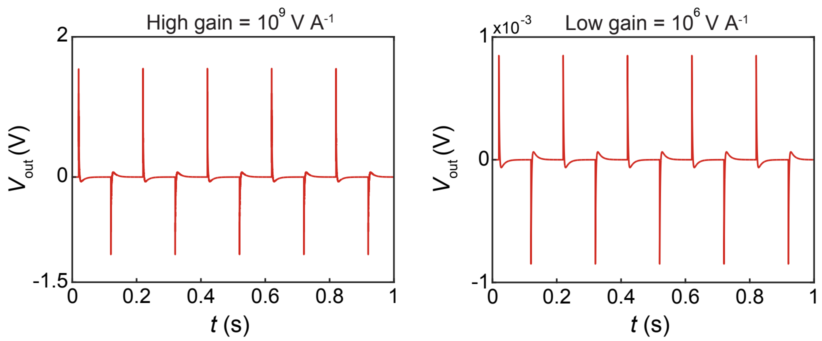

Supplementary Figure 8. TIA gain is found to affect the mismatch between $|A_{on}|$ and $|A_{off}|$ in V_{out} traces of a single CU. PDs are modeled the same way as Supplementary Fig. 9**b** (see below) with R_p = 100 GΩ, R_s = 100 kΩ, and C_j = 12 pF.

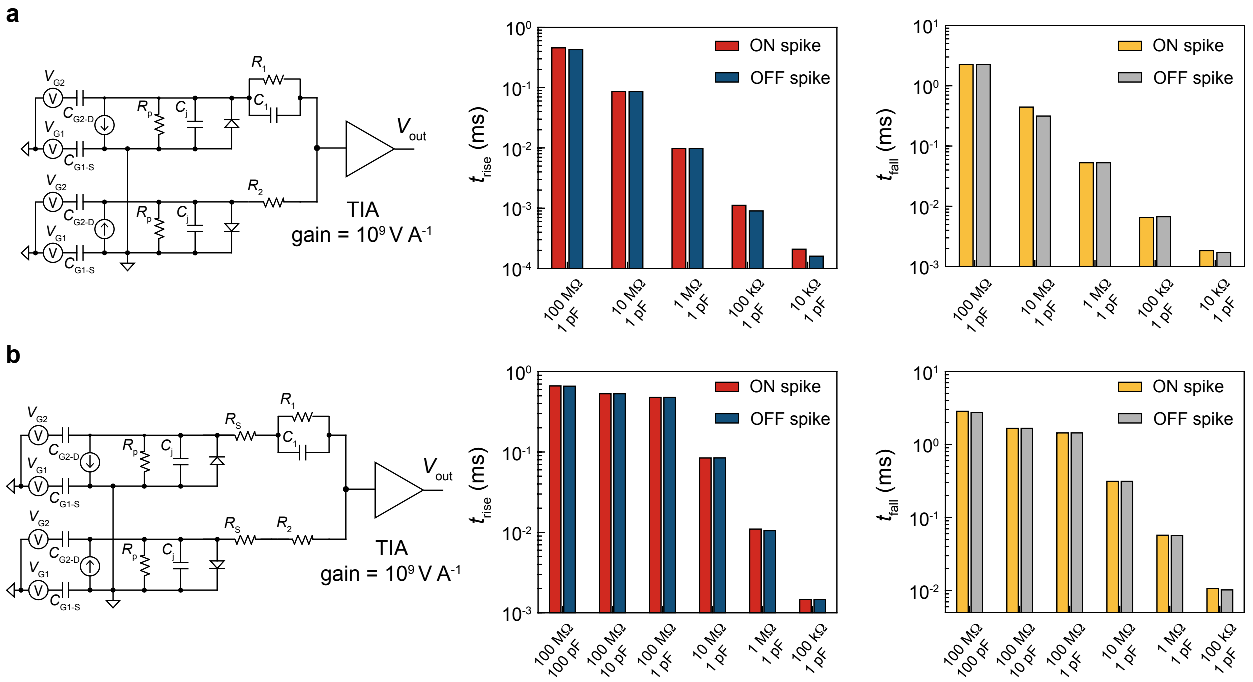

Supplementary Figure 9. LTspice simulation of t_{rise} and t_{fall} in a single CU with different values of the circuit parameters. a, Equivalent circuit and extracted t_{rise} and t_{fall} with R_p = 100 GΩ, R_s = 0 (neglected), and C_j = 12 pF. **b**, Equivalent circuit and extracted t_{rise} and $t_{fall.}$ with R_p = 100 GΩ, R_s = 100 kΩ, and C_j = 12 pF. Here we send current pulses to the ideal current source to emulate the photoresponse of a single PD; one 1 nA current pulse ($t_{on/off}$ = 50/50 ms) is configured to switch between its 10 and 90 % amplitude within 1 ns. In both **a** and **b**, we set V_{G1} = - V_{G2} = 2.5 V and $C_{G1\text{-}S}$ = $C_{G2\text{-}D}$ = 5 pF (estimated from our fabricated PDs).

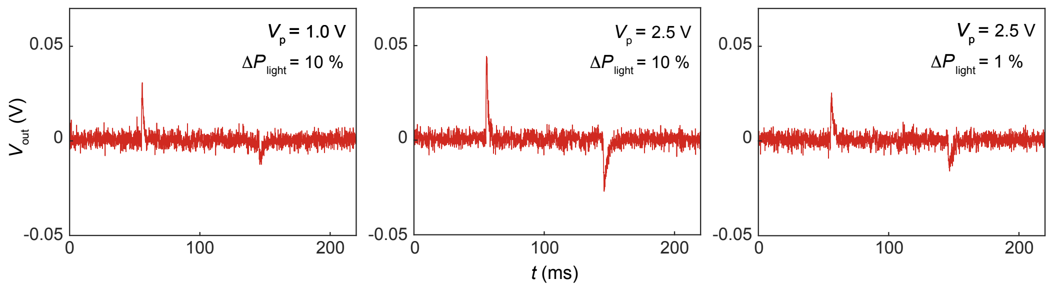

Supplementary Figure 10. CU output under various testing conditions. Representative V_{out} traces of the single CU tested in Fig. 2, whose signal-to-noise ratios vary with V_p and ΔP_{light} values.

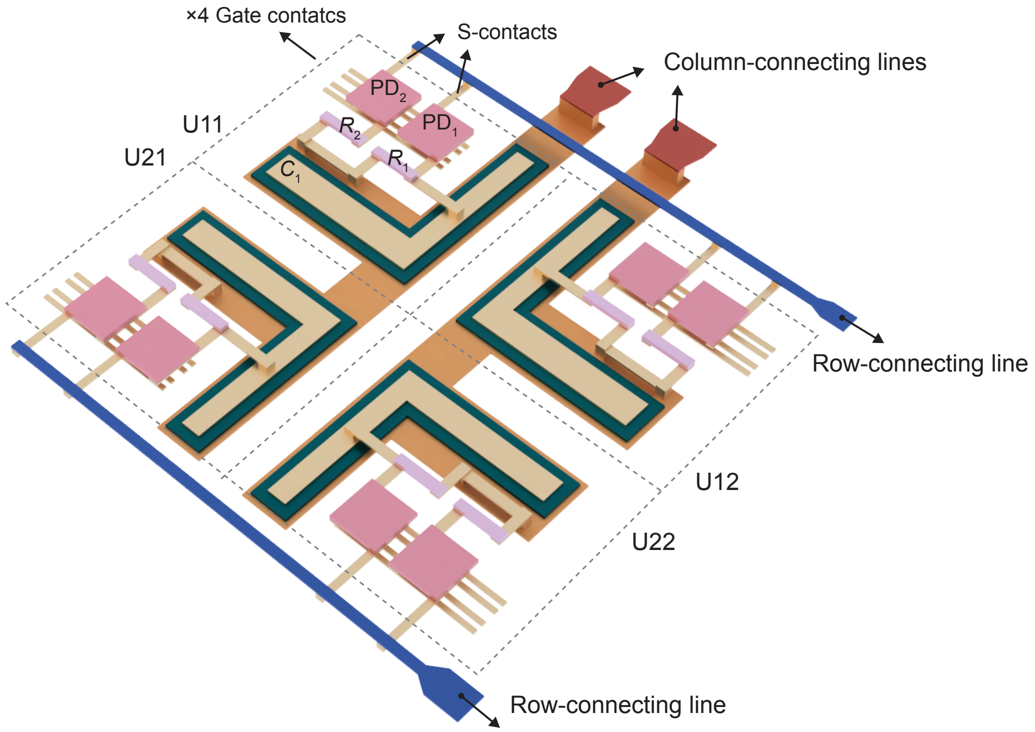

Supplementary Figure 11. 3D schematics of a CU array.

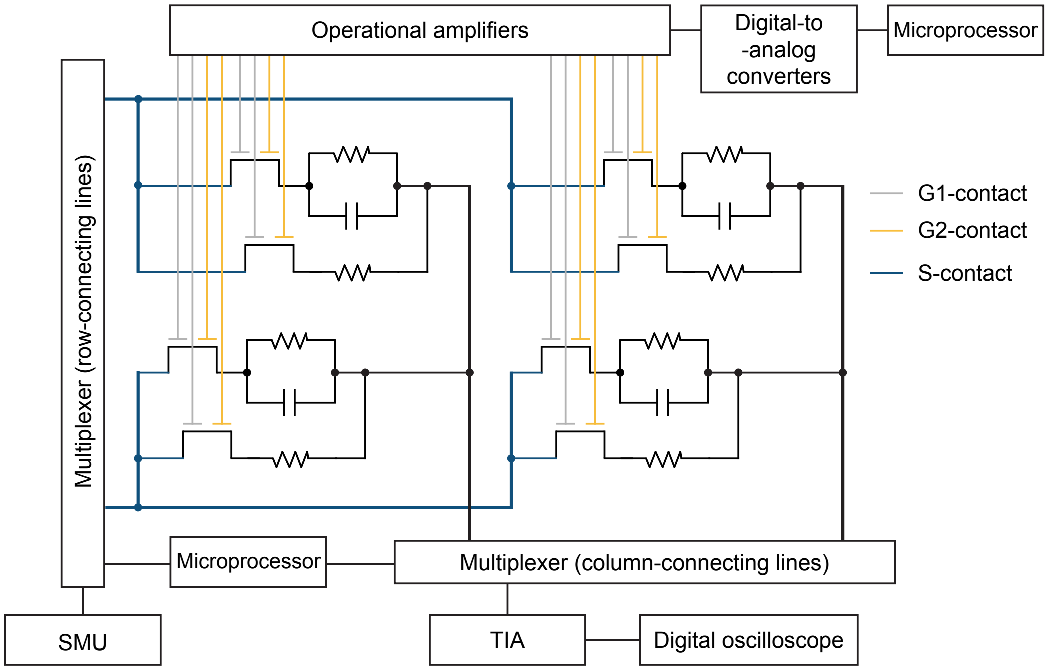

Supplementary Figure 12. Circuit diagram of the CU experiments.

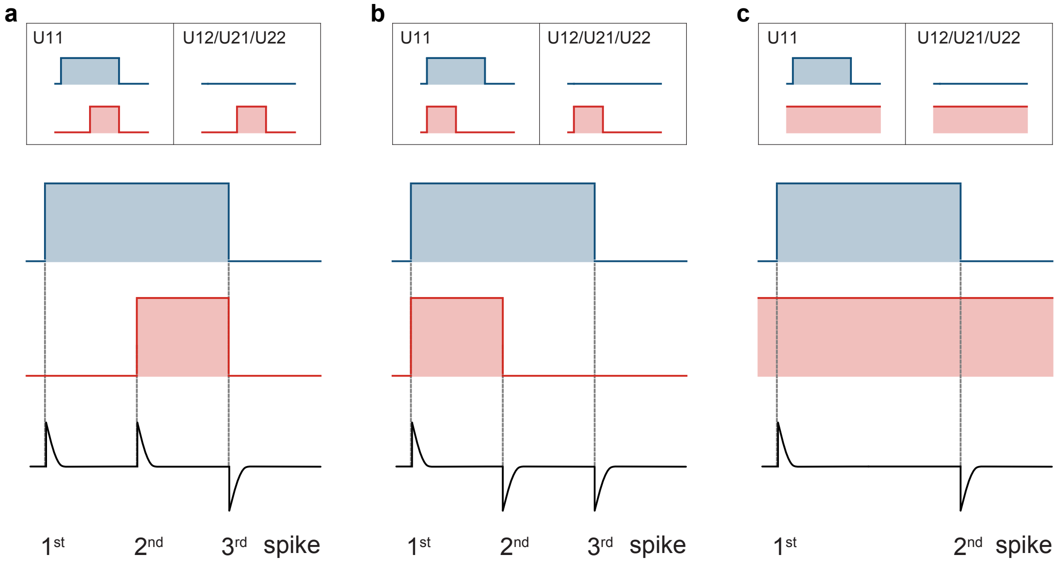

Supplementary Figure 13. Spike numbering of the CU array. **a-c**, Spikes numbered for illumination conditions I-III depicted in Fig. 3, respectively.

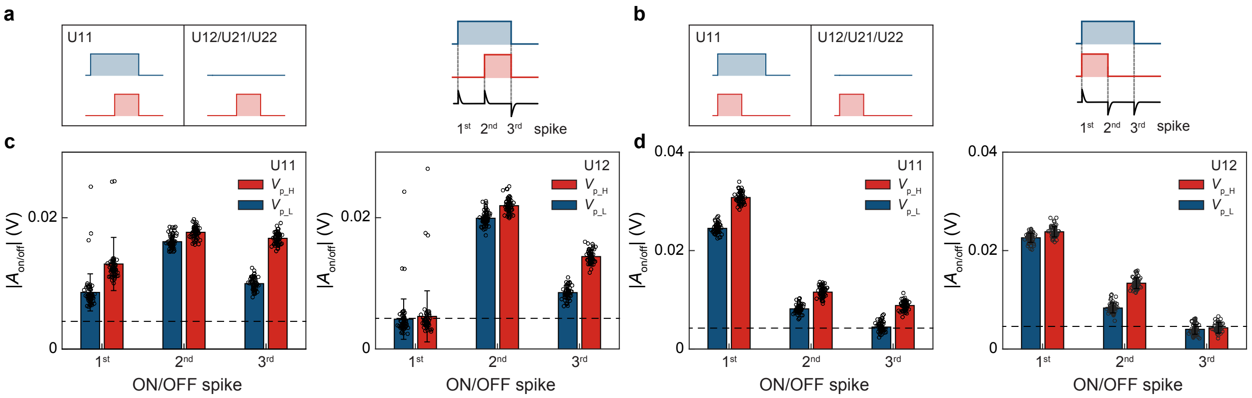

Supplementary Figure 14. Tuning output amplitudes of U11 and U12 by adjusting the V_p values. a, Spike numbering for illumination condition I. **b**, Spike numbering for illumination condition II. **c**, $|A_{on}|$ and $|A_{off}|$ values detected by U11 and U12 under condition I. **d**, $|A_{on}|$ and $|A_{off}|$ values detected by U11 and U12 under condition II. In **c** and **d**, 2 PDs in the CU are biased at V_{p_H} [V_{p_L}] to let each branch output V_{out} = ± 60 mV [40 mV] (see Supplementary Fig. 25); error bars represent ± 1 S.D. from a total of 60 pulses; dash lines represent the noise level (3 S.D.) calculated from the baseline data of each CU.

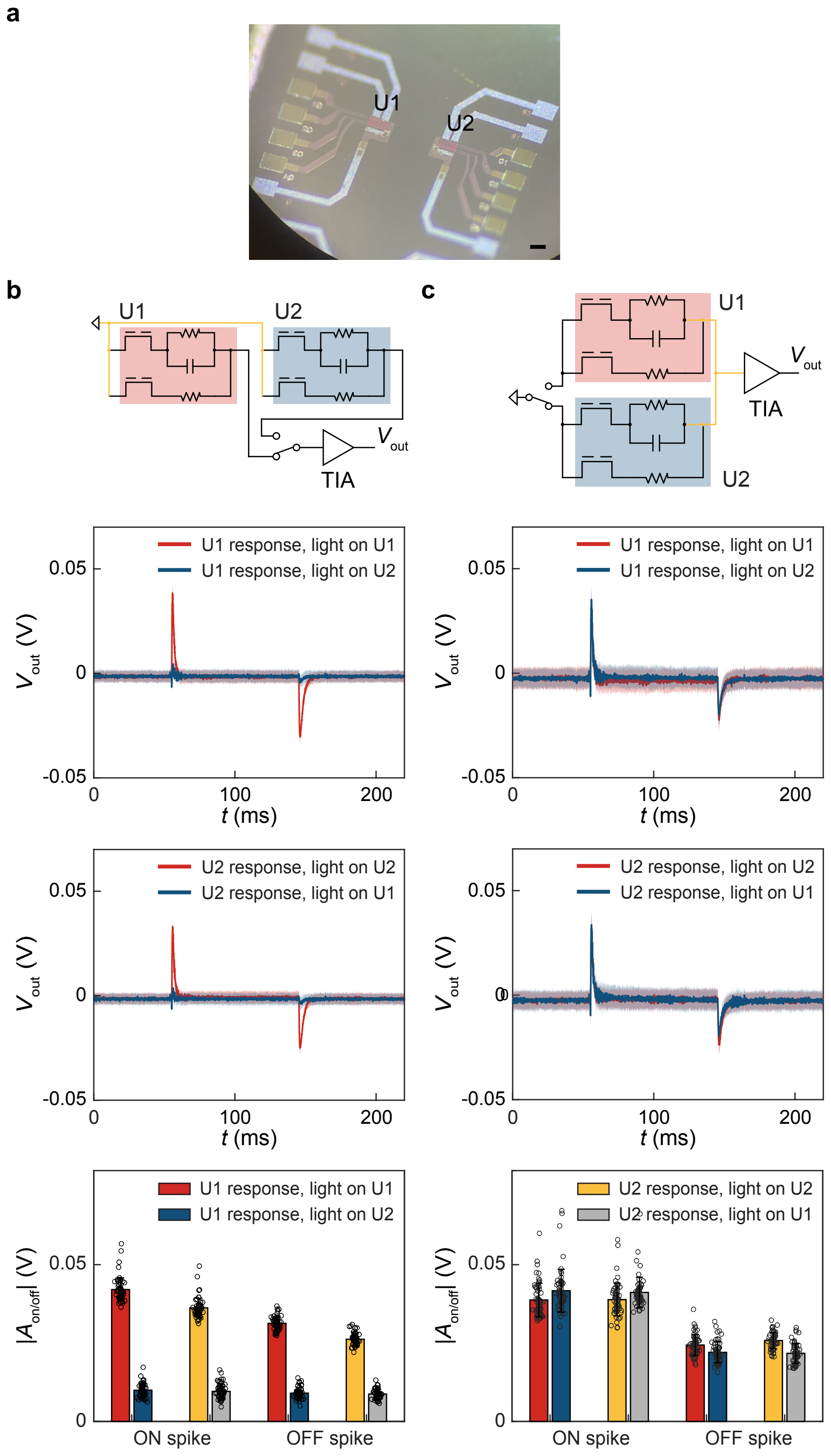

Supplementary Figure 15. Additional experiments on crosstalk analysis between CUs. a, Two separate CUs (U1, U2) on the same chip. Scale bar, 100 μm. **b-c,** $|A_{on}|$ and $|A_{off}|$ values detected by U1 and U2 when they are connected in the same way as U12 (**b**) and U21 (**c**) are connected to U11 in the array, respectively. Light pulses (ΔP_{light} = 530 mW cm^{-2} at 550/15 nm, t_{on}/t_{off} = 90/130 ms; three 20-pulse periods) are spatially confined to only U1 or U2. Shaded areas and error bars both represent ± 1 S.D. from a total of 60 pulses.

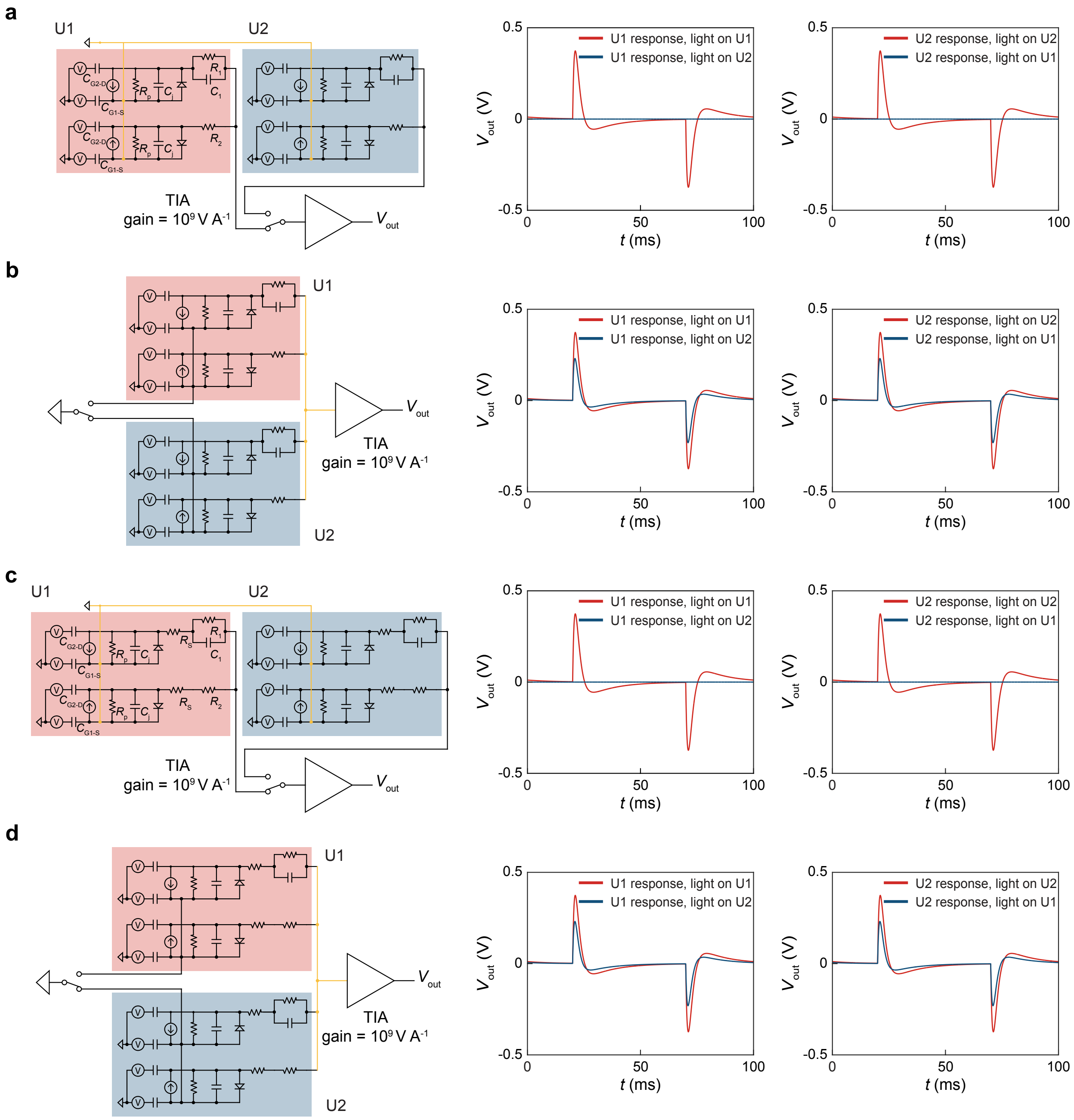

Supplementary Figure 16. LTspice simulation of the crosstalk from two connected CUs. PDs in **a** and **b** [**c** and **d**] are modeled the same way as Supplementary Fig. 9**a** [9**b**]. All PDs in **a-d** are biased at V_{G1} = -V_{G2} = 2.5 V; PDs under [without] light illumination are set with a 1 nA [0 nA] current pulse ($t_{on/off}$ = 50/50 ms) switching between its 10 and 90 % amplitude within 1 ns.

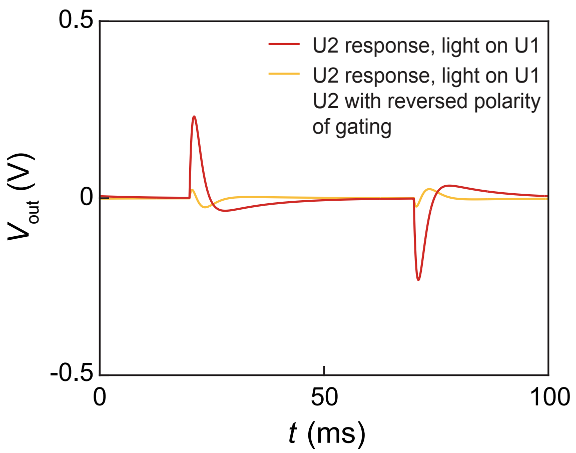

Supplementary Figure 17. LTspice simulation of the crosstalk mitigation in Supplementary Fig. 16d. PDs in U1 and U2 are modeled the same way as Supplementary Fig. 9. In the red curve, PDs in U1 [U2] are biased at V_{G1} = - V_{G2} = 2.5 V and a 1 nA [0 nA] current pulse ($t_{on/off}$ = 50/50 ms) switching between its 10 and 90 % amplitude within 1 ns. In the yellow curve, PDs in U1 are biased the same as before but those in U2 are biased at V_{G1} = - V_{G2} = -1.4 V and a -0.56 nA current pulse ($t_{on/off}$ = 50/50 ms) switching between its 10 and 90 % amplitude within 1 ns (note: the current value matches those measured from our fabricated PDs). Our data show that the crosstalk at non-targeted U2 (with light illumination on U1 only) can be significantly reduced if we reverse the polarity of its gate biases.

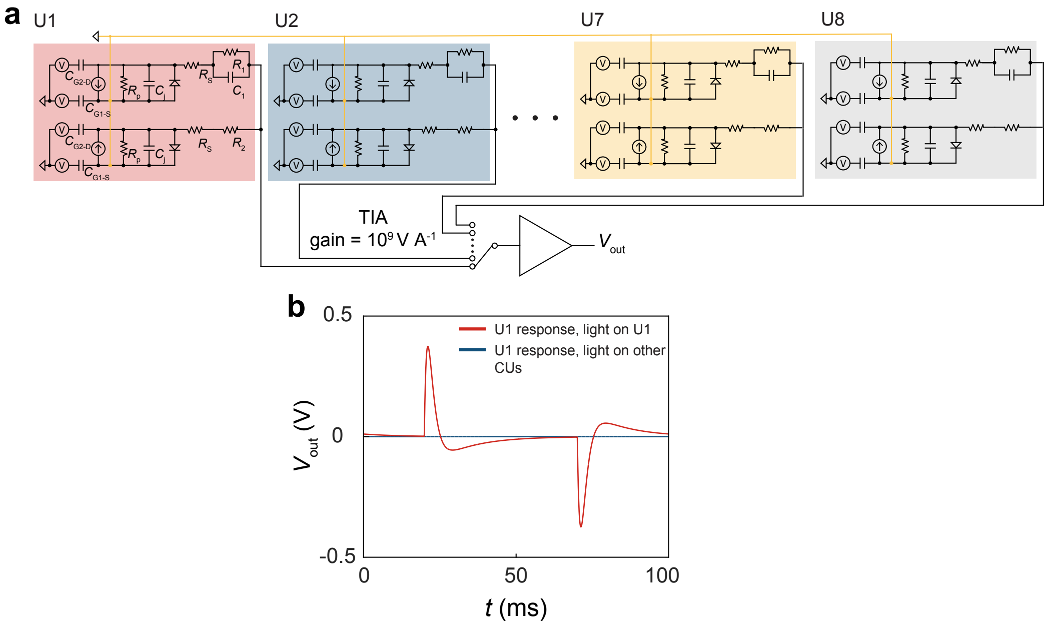

Supplementary Figure 18. LTspice simulation of the crosstalk from a 1-by-8 CU array. PDs in U1-U8 are modeled the same way as Supplementary Fig. 9.

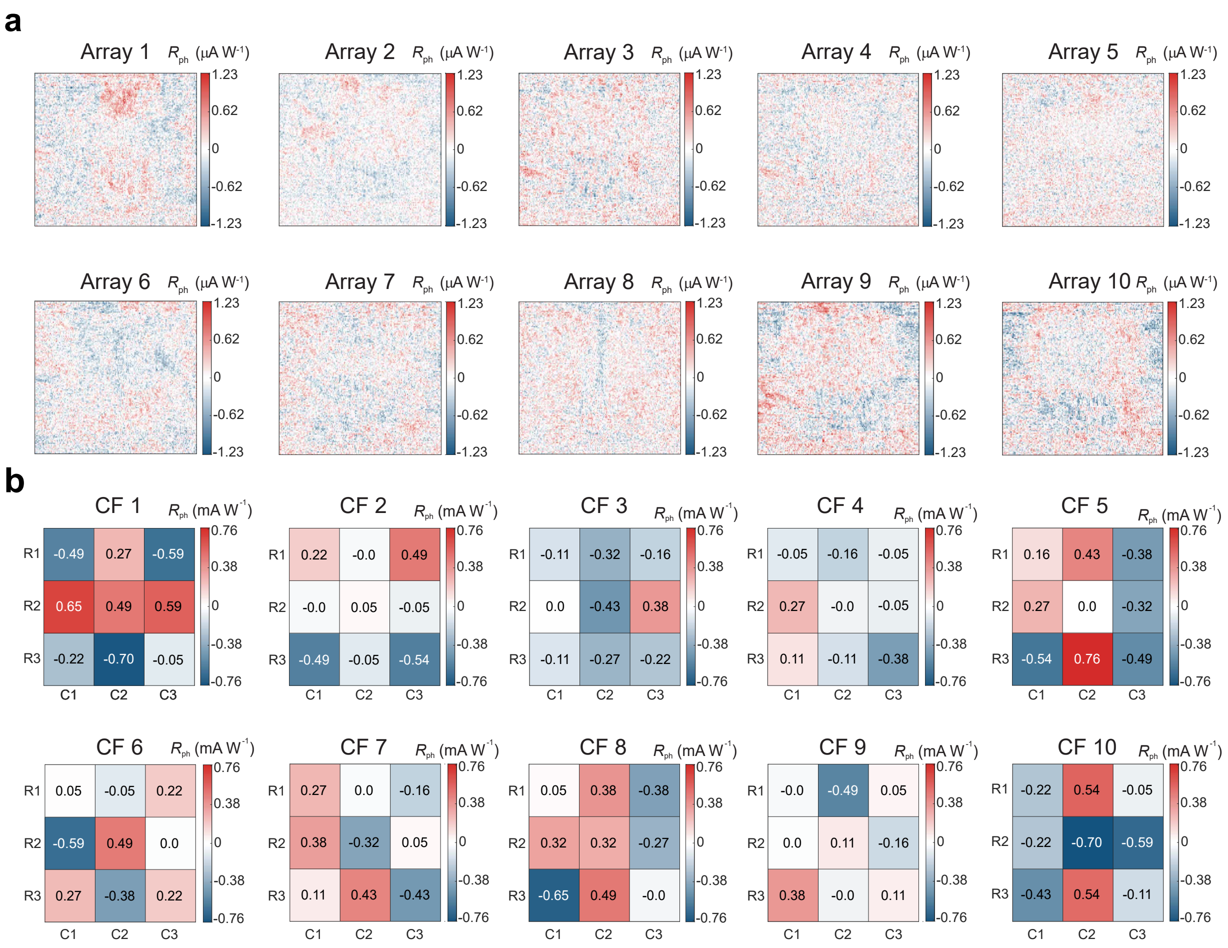

Supplementary Figure 19. R_{ph} values determined by normalizing learned weights from the SNN/ANN training. a, R_{ph} values of the PD in the 1R1C branch (opposite to those in the 1R branch) across ten 120 × 160 CU arrays. **b,** R_{ph} values of 10 CFs (each of which is a 3-by-3 PD array) in an 8-by-8 kernel array. R1-3 and C1-3 represent row and column positions of the PD in the kernel, respectively.

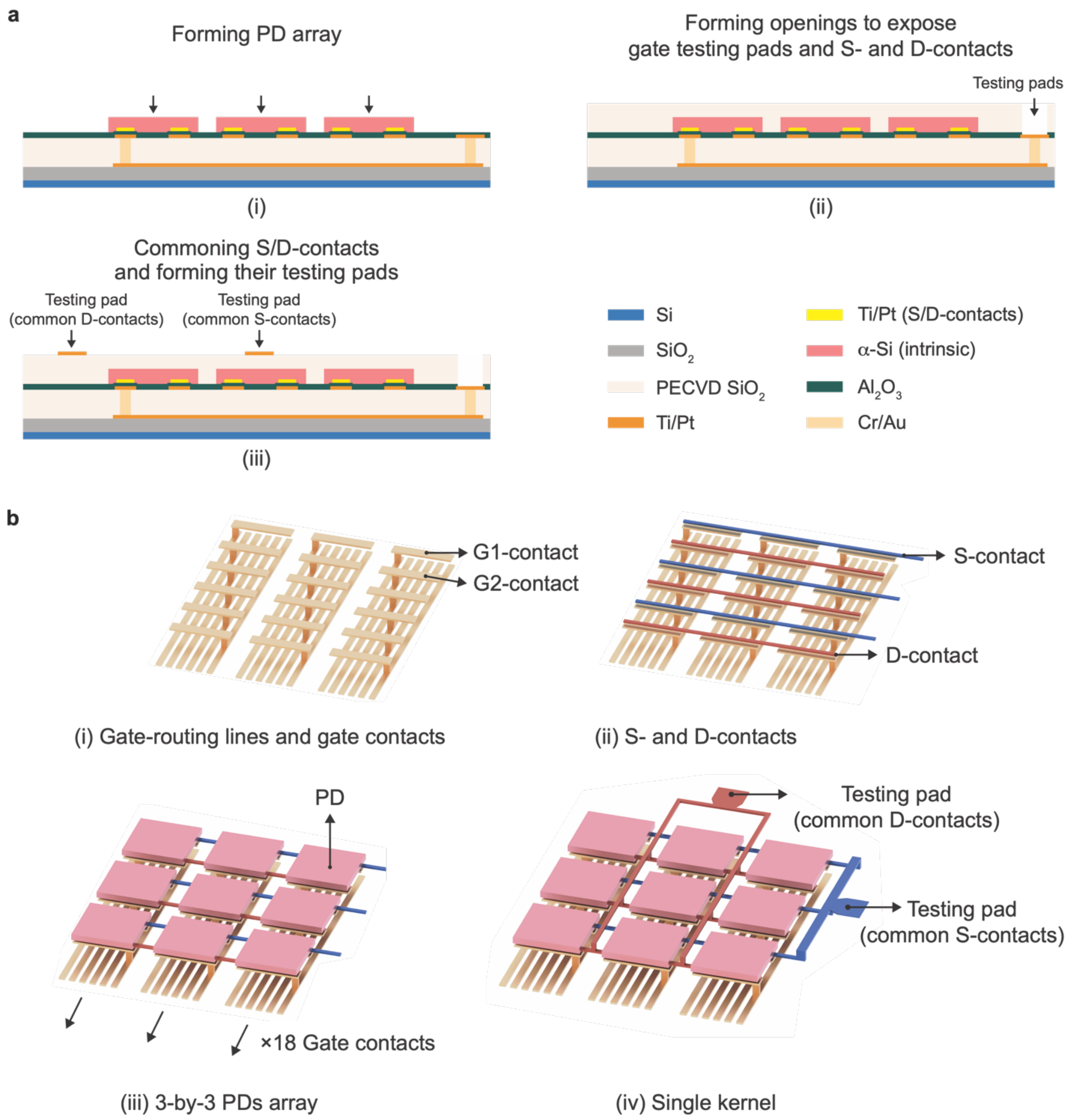

Supplementary Figure 20. Fabrication of single kernels. a, Fabrication flow. **b**, 3D schematics.

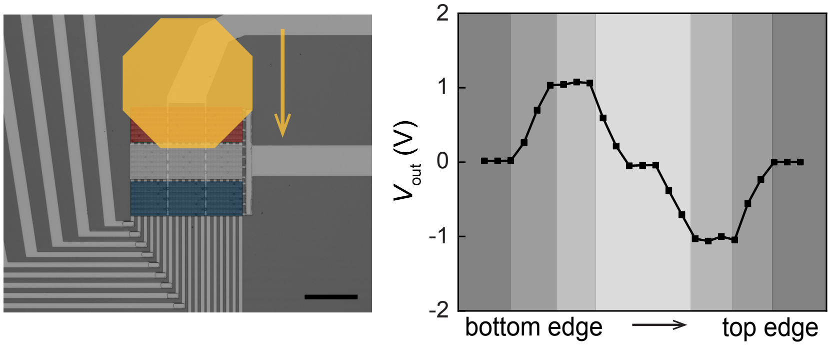

Supplementary Figure 21. Additional experiments on edge detection with single kernels. A kernel configured as a vertical Prewitt filter (see V_p values in Supplementary Tab. 2) is used to detect the edges of a vertically moving light spot (P_{light} = 530 mW cm^{-2} at 550/15 nm) based on V_{out} values measured at a *ca.* 23 µm step. Scale bars, 100 µm.

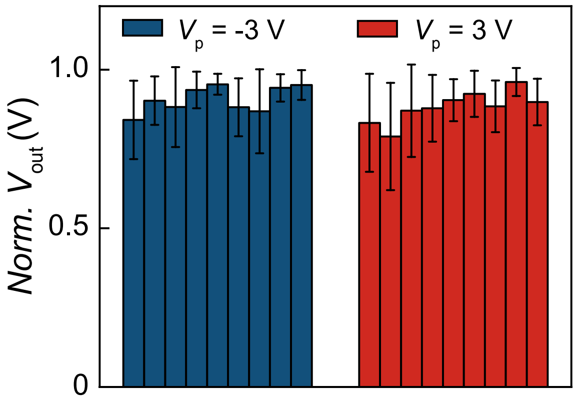

Supplementary Figure 22. PD readout at V_p = -3 V and 3 V from four kernels in the center of a kernel array. Bars represent 9 PDs in each kernel; error bars represent ± 1 S.D. from four kernels.

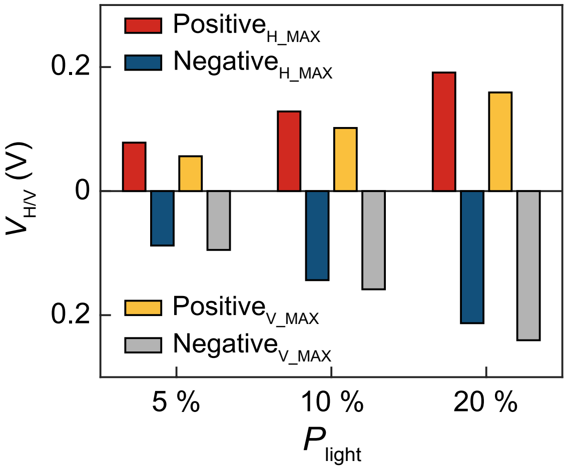

Supplementary Figure 23. P_{light}-dependence of the readout of a kernel array configured for in-sensor edge detection. Experiments in Fig. 5**g** are repeated with P_{light} = 265 mW cm^{-2} (5 %), 530 mW cm^{-2} (10 %), and 1060 mW cm^{-2} (20 %) at 550/15 nm. Bars represent the positive- [negative-] maximum readout in each $V_{H/V}$ heat map.

a

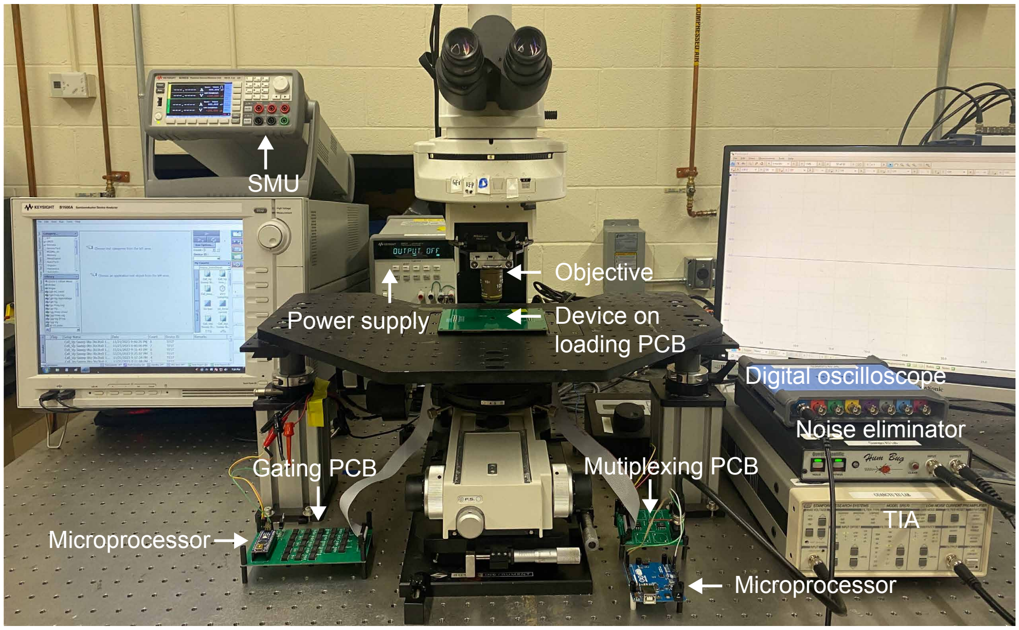

b

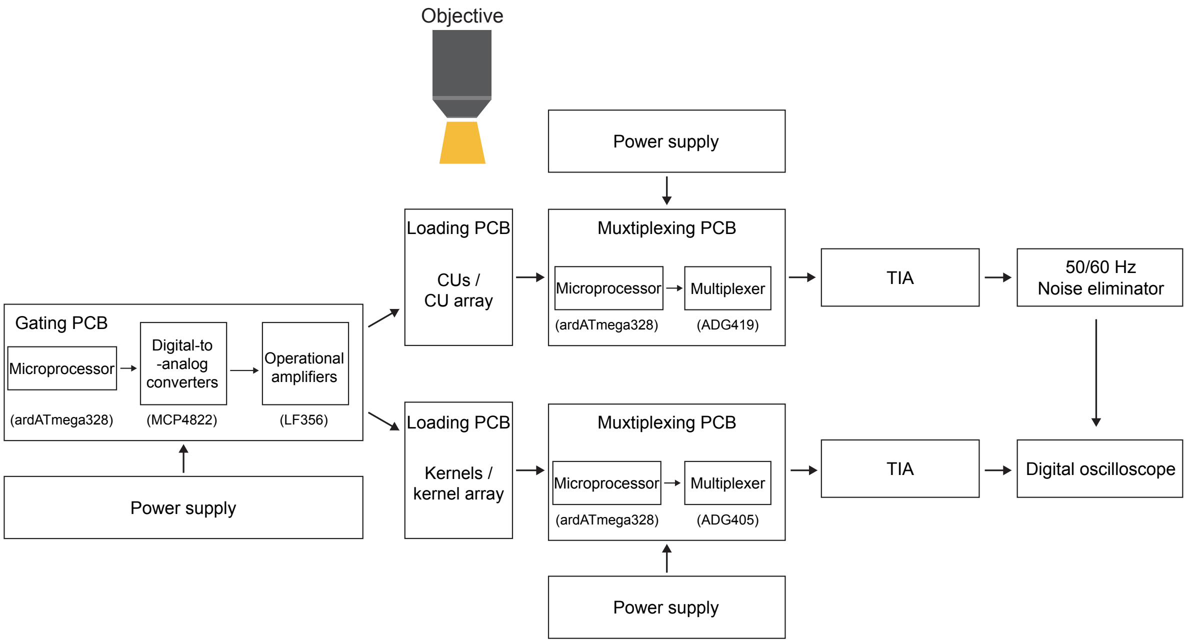

Supplementary Figure 24. Experimental setup. **a**, Exemplified testing setup for single CUs and the CU array. **b**, Block diagrams of the testing setups.

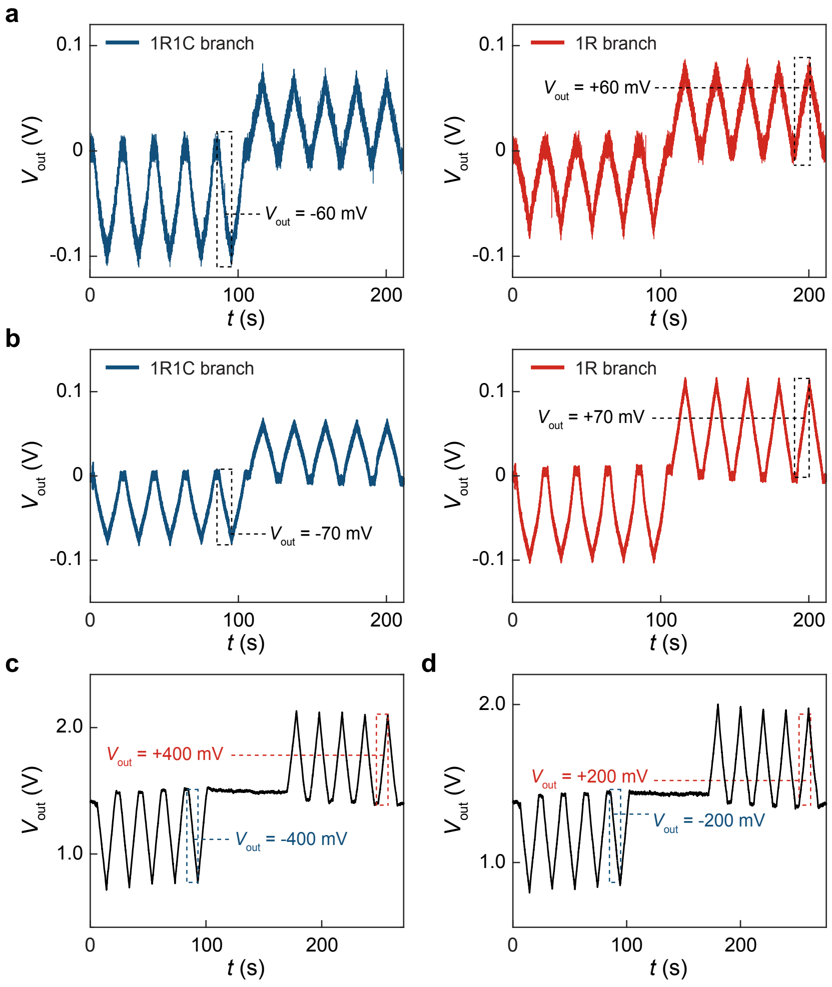

Supplementary Figure 25. Deciding the value of V_p needed to output targeted V_{out} values at the steady state. V_p-dependence of V_{out} values from a CU (**a**), a representative CU in a CU array (**b**), a representative PD from a kernel (**c**), and a representative PD from a kernel array (**d**). For each branch, we first sweep V_p for five consecutive 0 → -3 → 0 V cycles, followed by five consecutive 0 → + 3 → 0 V cycles. The squared half cycle (in which we believe the PD has reached to its steady state) is chosen to decide the value of V_p needed to output targeted V_{out} values (e.g., (**a**) V_{out} = ± 60 mV in Supplementary Fig. 7, (**b**) V_{out} = ± 70 mV in Fig. 3, (**c**) V_{out} = ± 400 mV in Fig. 5**d**, (**d**) V_{out} = ± 200 mV in Figs. 5**g** and **h**). The light spot (P_{light} = 530 mW cm^{-2} at 550/15 nm) is spatially confined to the PD in the select branch (**a**-**b**), and illuminating the whole kernel/kernel array (**c**-**d**).

Event detection with a single CU (unit: V)

	V_{out} (P_{light} = 530 mW cm^{-2}, 550/15 nm)	PD_1	PD_2
Fig. 2**c**	± 60 mV	-2.75	3.00
Figs. 2**d**, **e** and **f**	± 10 mV	-1.09	0.99
	± 20 mV	-1.33	1.37
	± 30 mV	-1.70	1.84
	± 40 mV	-1.96	2.16
	± 50 mV	-2.24	2.50
Figs. 2**g**, **h** and **i**	± 60 mV	-2.75	3.00
Supplementary Fig.7	± 60 mV	-2.75	3.00

Event detection with a CU array (unit: V)

	CU in the array	PD_1		PD_2	
Fig. 3	U11	-2.74		2.03	
	U21	-1.80		2.89	
	U12	-2.66		3.00	
	U22	-2.69		2.85	
		V_{P_H}	V_{P_L}	V_{P_H}	V_{P_L}
Supplementary Fig.12	U11	-2.39	-1.12	3.00	1.20
	U21	-2.58	-1.02	3.13	1.45
	U12	-2.78	-0.89	2.95	1.36
	U22	-2.62	-1.10	2.85	1.60

Crosstalk in a CU array (unit: V)

		PD_1	PD_2
Supplementary Fig.13	U1	-2.67	2.98
	U2	-2.76	2.70

Supplementary Table 1. V_p values used in the CU experiments.

<table>
<tr><th colspan="6">Edge detection with a single kernel (unit: V)</th></tr>
<tr><td></td><td></td><td></td><td>C1</td><td>C2</td><td>C3</td></tr>
<tr><td rowspan="3">Fig. 5c</td><td rowspan="3">Horizontal
Prewitt filter</td><td>R1</td><td>-2.80</td><td>0.10</td><td>2.30</td></tr>
<tr><td>R2</td><td>-2.80</td><td>0.20</td><td>2.30</td></tr>
<tr><td>R3</td><td>-2.20</td><td>0.10</td><td>2.30</td></tr>
<tr><td></td><td></td><td></td><td>C1</td><td>C2</td><td>C3</td></tr>
<tr><td rowspan="3">Supplementary Fig. 16</td><td rowspan="3">Vertical
Prewitt filter</td><td>R1</td><td>2.50</td><td>2.30</td><td>2.30</td></tr>
<tr><td>R2</td><td>0.10</td><td>0.20</td><td>0.10</td></tr>
<tr><td>R3</td><td>-2.10</td><td>-2.20</td><td>-2.00</td></tr>
<tr><th colspan="6">Edge detection with a kernel array (unit: V)</th></tr>
<tr><td></td><td></td><td></td><td>C1</td><td>C2</td><td>C3</td></tr>
<tr><td rowspan="8">Figs. 5g and h
Supplementary Fig. 19</td><td rowspan="3">Horizontal
Prewitt filter</td><td>R1</td><td>0.90</td><td>0.00</td><td>-1.20</td></tr>
<tr><td>R2</td><td>0.70</td><td>0.00</td><td>-1.30</td></tr>
<tr><td>R3</td><td>0.03</td><td>0.00</td><td>-1.10</td></tr>
<tr><td></td><td></td><td>C1</td><td>C2</td><td>C3</td></tr>
<tr><td rowspan="3">Vertical
Prewitt filter</td><td>R1</td><td>-1.10</td><td>-1.20</td><td>-1.10</td></tr>
<tr><td>R2</td><td>0.10</td><td>0.10</td><td>0.10</td></tr>
<tr><td>R3</td><td>0.06</td><td>0.70</td><td>1.10</td></tr>
</table>

Supplementary Table 2. V_p values used in the kernel experiments. R1-3 and C1-3 represent row and column positions of the PD in the kernel, respectively (see Supplementary Fig. 19).

Ref.	Pixel size (μm)	FF (%)	Reported/expected events per second (i.e. eps)
1	4.95 × 4.95	22	1.3 G
2	9 × 9	-	300 M
3	2.97 × 2.97	-	1.412 G
4	40 × 40	9.4	66 K (15 μs latency)
5	18.5 × 18.5	22	50 M
6	4.86 × 4.86	77	1.066 G
7	3 × 15	-	200 K (5 μs temporal resolution)
This work	83 × 83	> 30	*ca.*1 K in Fig. 2, 100 K – 1M if built with smaller *RC*s in Supplementary Fig. 9

Supplementary Table 3. Event detection using CMOS imagers and CU arrays (this work).

Ref.	Array dimension	Pixel size (μm)	FF (%)
8	128 × 128	7.6 × 7.6	36
9	160 × 120	4.9 × 4.9	53
10	105 × 92	8 × 8	11.69
11	80 × 78	45.6 × 45, 9.5 × 9.5	33
12	320 × 320	8 × 8	-
13	512 × 384	9.3 × 9.3	24
14	64 × 64	35 × 35	23
15	32 × 32	21 × 18.75	35.6
16	1920 × 1440	1.4 × 1.4	52.55
This work	24 × 24	76 × 76	> 90

Supplementary Table 4. Edge detection using CMOS imagers and kernel arrays (this work).

Supplementary References:

1. Suh, Y. *et al*. A 1280× 960 dynamic vision sensor with a 4.95-µm pixel pitch and motion artifact minimization. *2020 IEEE international symposium on circuits and systems (ISCAS)*. Seville, Spain, pp. 1-5, IEEE, 2020.
2. Son, B. *et al*. 4.1 A 640× 480 dynamic vision sensor with a 9µm pixel and 300Meps address-event representation. *2017 IEEE International Solid-State Circuits Conference (ISSCC).* San Francisco, CA, USA, pp. 66-67, IEEE, 2017.
3. Niwa, A. *et al*. A 2.97 µm-pitch event-based vision sensor with shared pixel front-end circuitry and low-noise intensity readout mode. *2023 IEEE International Solid-State Circuits Conference (ISSCC).* San Francisco, CA, USA, pp. 4-6, IEEE, 2023.
4. Lichtsteiner, P., Posch, C., & Delbruck, T. A 128×128 120 dB 15 µs latency asynchronous temporal contrast vision sensor. *IEEE J. of solid-state circuits* **43** 566-576 (2008).
5. Brandli, C. *et al*. A 240× 180 130 db 3 µs latency global shutter spatiotemporal vision sensor. *IEEE J. of Solid-State Circuits* **49** 2333-2341 (2014).
6. Finateu, T. *et al*. 5.10 a 1280× 720 back-illuminated stacked temporal contrast event-based vision sensor with 4.86 µm pixels, 1.066 GEPS readout, programmable event-rate controller and compressive data-formatting pipeline. *2020 IEEE International Solid-State Circuits Conference-(ISSCC)*. San Francisco, CA, USA, pp. 112-114, IEEE, 2020.
7. Zhou, Y. *et al*. Computational event-driven vision sensors for in-sensor spiking neural networks. *Nat. Electron*. 6, 1-9 (2023).
8. Hsu, T.H. *et al*. A 0.5-V real-time computational CMOS image sensor with programmable kernel for feature extraction. *IEEE J. of Solid-State Circuits* **56**, 1588-1596 (2020).
9. Kim, H.J. *et al*. A dual-imaging speed-enhanced CMOS image sensor for real-time edge image extraction. *IEEE J. of Solid-State Circuits* **52,** 2488-2497 (2017).

10. Lee, C., Chao, W. *et al*. A low-power edge detection image sensor based on parallel digital pulse computation. *IEEE Trans. on Circuits and Systems II: Express Briefs* **62**, 1043-1047 (2015).

11. Etienne-Cummings, R., Kalayjian, Z. K., & Cai, D. A programmable focal-plane MIMD image processor chip. *IEEE J. of Solid-State Circuits* **36** 64-73 (2001).

12. Park, M. J., & Kim, H. J. A Real-Time Edge-Detection CMOS Image Sensor for Machine Vision Applications. *IEEE Sensors Journal* **23** 9254-9261 (2023).

13. Muramatsu, Y., Kurosawa, S. *et al*. A signal-processing CMOS image sensor using a simple analog operation. *IEEE J. of Solid-State Circuits* **38** 101-106 (2003).

14. Jendernalik, W., Blakiewicz *et al*. An analog sub-miliwatt CMOS image sensor with pixel-level convolution processing. *IEEE Trans. on Circuits and Systems I: Regular Papers* **60** 279-289 (2013).

15. Nazhamaiti, M. *et al*. NS-MD: Near-sensor motion detection with energy harvesting image sensor for always-on visual perception. *IEEE Trans. on Circuits and Systems II: Express Briefs* **68** 3078-3082 (2021).

16. Jin, M. *et al*. Design of an edge-detection cmos image sensor with built-in mask circuits. *Sensors* **20** 3649 (2020).
